# Mortuary Report

**Published:** 1881-05

**Authors:** 


### MARCH REPORT OF DEATHS AND INTERMENTS IN BALTIMORE.

| CAUSE OF DEATH. | No. | CAUSE OF DEATH. | No. | CAUSE OF DEATH. | No. |
|---|---|---|---|---|---|
| Abscess | 2 | Disease of liver | 2 | Marasmus | 19 |
| Adynamia | 1 | " spine | 1 | Metro peritonitis | 1 |
| Anamia | 1 | Dropsy, (gen'l) | 3 | Measles | 10 |
| Aortic regurgitation | 1 | " scarlatinal | 1 | Menorrhagia | 1 |
| Aneurism of aorta | 1 | Drowned | 1 | Meningitis | 17 |
| " innominate artery | 1 | Dysentery | 1 | " Tubercular | 6 |
| Angina pectoris | 1 | Entero colitis | 1 | " cerebro spinal | 6 |
| Albuminuria | 1 | Endocarditis | 1 | Murder | 1 |
| Apoplexy | 9 | Erysipelas | 10 | Multiple sarcoma | 1 |
| Addison's disease | 1 | Eczema | 1 | Osteo sarcoma | 1 |
| Ascites | 1 | Enlargement of liver | 1 | Old Age | 27 |
| Asthma | 2 | Fever, malarial | 1 | Paralysis | 22 |
| Asthenia | 6 | " intermittent | 1 | Pneumonia | 56 |
| Atelectasis pulmonum | 1 | " remittent | 1 | " broncho | 3 |
| Bright's disease | 14 | " rheumatic | 2 | " typhoid | 2 |
| Burns | 4 | " scarlet | 21 | " pleuro | 3 |
| Capillary bronchitis | 5 | " typhoid | 9 | Poison | 2 |
| Carbuncle | 1 | " Typhus | 1 | Premature birth | 15 |
| Cancer, diffused | 1 | " typho-malarial | 2 | Phthisis laryngeal | 1 |
| " mouth | 1 | Fracture, leg | 1 | Purpura | 1 |
| " breast | 5 | " skull | 3 | Phrenitis | 1 |
| " liver | 1 | Gastro enteritis | 3 | Puerpural septicæmia | 1 |
| " pylorus | 1 | Hernia, strangulated | 1 | Pyæmia | 2 |
| " uterus | 7 | Hemorrhage, bowels | 2 | Rheumatism, inflammatory | 3 |
| " vagina | 1 | " lungs | 2 | " heart | 5 |
| " stomach | 5 | Hydrocephalus | 3 | Scrofula | 5 |
| Child birth | 1 | Inanition | 10 | Softening of brain | 3 |
| Congestion of brain | 8 | Imperfect development | 1 | Septicæmia | 1 |
| " lungs | 4 | Inflammation of brain | 3 | Scalds | 1 |
| Consumption of Lungs | 122 | " bladder | 3 | Suicide | 3 |
| Convulsions | 36 | " bowels | 2 | Syphilis | 3 |
| Compression of brain | 1 | " stomach | 8 | Tetanus | 2 |
| Cholera | 1 | " bronchi | 12 | Ulcer, chronic | 1 |
| Croup | 17 | Nervous prostration | 2 | Unknown Adult | 3 |
| Cyanosis | 1 | " kidneys | 3 | " Infantile | 7 |
| Cholic | 2 | " larynx | 1 | Whooping Cough | 7 |
| Debility | 2 | " liver | 4 | Wounds | 2 |
| Dentition | 7 | " pericard'm. | 1 | Extra Uterine Pregnancy | 2 |
| Delirium tremens | 1 | " peritoneum | 6 | Progressive Locomatix Atoxica | 1 |
| Dyspepsia | 1 | " pleura | 1 | Specific Consumption | 1 |
| Diarrhœa | 3 | Intemperance | 3 | | |
| Diphtheria | 25 | Jaundice | 1 | | |
| Disease of heart | 32 | Malnutrition | 1 | Total | 714 |

THE DEATHS REGISTERED IN THE MONTH INCLUDE

| | | | |
|---|---|---|---|
| Under 1 year | 154 | Between 20 and 30 " | 56 |
| Between 1 and 2 years | 57 | " 30 and 40 " | 72 |
| " 2 and 5 years | 52 | " 40 and 50 " | 47 |
| | | " 50 and 60 " | 55 |
| Under 5 years | 263 | " 60 and 70 " | 65 |
| Between 5 and 10 years | 49 | " 70 and 80 " | 55 |
| " 10 and 15 " | 9 | " 80 and 90 " | 22 |
| " 15 and 20 " | 18 | " 90 and 100 " | 3 |

NATIVITY, ETC.

| | |
|---|---|
| United States—White | 414 |
| " Colored | 178 |
| Foreign | 122 |
| Total for the month | 714 |
| Still Birth | 69 |

Among the decedents were 85 above the age of 70 years, whose combined ages were 6,643 years, averaging 77 years 8 months.

Estimated Population, 393,796. white, 338,384. Colored, 55,412. Annual Death Rate during the month, whole population, per 1,000—21.74. White, 19.02. Colored, 38.83.
Births reported, 650.

By Order: JAMES A. STEUART, M. D., Commissioner of Health and Registrar.

A. R. CARTER, Secretary.

---

U. S. SIGNAL OFFICE, BALTIMORE, MD., April 1, 1881.

### METEOROLOGICAL SUMMARY FOR THE MONTH OF MARCH, 1881.

### PRESSURE.

Mean Monthly Barometer, - - - - - 29.800 inches.
" " " for past 10 years, - -
Highest Barometer, - - - - - - - 30.395 " on the 15th.
Lowest " - - - - - - - 29.036 " on the 30th.
Monthly Range of Barometer, - - - - - 1.359 "

### TEMPERATURE.

Mean Monthly Thermometer, - - - - - 42.0 degrees.
" " " for past 10 years - "
Highest Thermometer, - 59 degrees on 16th. Monthly Range – 32 degrees.
Lowest " - 27 degrees on 1st & 2d. Mean relative humidity, 63.2 "

### WIND, ETC.

Prevailing direction of wind, west. Total amount of rainfall, inches.
Highest velocity of wind, 24 miles per hour. Total movement of wind, 6,595 miles.
Number of days on which rain fell, 14. Total rainfall, 6.68 inches.

ROBERT SEYBOTH, in charge.

